# The Regulatory Roles of MicroRNA in Effects of 2,2'4,4'-Tetrabromodiphenyl Ether (BDE47) on the Transcriptome of Zebrafish Larvae

**DOI:** 10.1371/journal.pone.0169599

**Published:** 2017-01-10

**Authors:** Jing Zhao, Ting Xu, Daqiang Yin, Bo Zhang, Jianfeng Bai

**Affiliations:** 1 Shanghai Collaborative Innovation Centre for WEEE Recycling, WEEE Research Centre of Shanghai Polytechnic University, Shanghai, China; 2 Key Laboratory of Yangtze River Water Environment, Ministry of Education, College of Environmental Science and Technology, Tongji University, Shanghai, China; 3 Novel Bioinformatics Co., Ltd, Shanghai, China; East Carolina University, UNITED STATES

## Abstract

The developmental neurotoxicity caused by environmental pollutants has received great concern; however, there were still barely known about the underlying toxic mechanisms, especially the influence of varieties of regulatory factors such as microRNA (miRNA). A representative flame retardant, 2,2′,4,4′-tetrabromodiphenyl ether (BDE47), was found to disrupt zebrafish development in visual perception and bone formation in previous study, thus here we investigated its effects on miRNA expression profiling of 6 days post fertilization (dpf) zebrafish larvae by deep sequencing. To overcome the shortage of zebrafish miRNA annotation, multiple data processing approaches, especially constructed network based on the interactions between miRNAs and enrichment terms, were adopted and helped us acquire several validated zebrafish miRNAs and two novel miRNAs in BDE47-induced effects, and identify corresponding biological processes of the miRNAs. Among them, miR-735 was supposed to play essential roles in larval sensory development according to analysis results. Our study also provided an effective strategy for analyzing biological effects on non-mammalian miRNAs with limited basic information.

## Introduction

MicroRNAs (miRNAs) are an abundant class of 21–24 nucleotide noncoding RNAs that function as negative regulators of posttranscriptional gene expression by slicing mRNAs or inhibiting the translation of mRNAs [1,2]. Since the first miRNA, lin4, was found in 1993 [3], the regulatory functions of miRNA were hoped to interpret the gene expression changes under intrinsic program or exogenous stimuli. At least 30% of genes are predicted to be regulated by miRNAs, and the impacts involved diverse biological processes of yeasts, protozoans, plants, and animals [4–6]. Notably, fundamental functional studies of animal miRNAs other than human and rodents were still relatively limited. This state caused inconvenience to miRNA studies on other species, and was in part contributed to that the interests in miRNA were mainly from research on human diseases, particularly cancer [7,8].

Development was another event which miRNA was deeply participated in. Accumulating evidences showed exposure to environmental pollutants disrupted normal developmental processes of wildlife. Taken rodent as example, prenatal and neonatal exposure to perfluorooctane sulfonate (PFOS) caused changes in miRNA expression profiles in developing rat brains, which could partly be responsible for persistent effects such as neurobehavioral dysfunctions [9]. Given that brain morphogenesis disruption by ethanol exposure resulted in abnormal neurobehavioral development in zebrafish larvae and juveniles, miRNAs were essential for the establishment of vertebrate neurobehavioral and skeletal systems (e.g., miR-9/9* and miR-153c) [10]. Such studies were similar to miRNA signatures/fingerprints in cancer research, however, they often stuck on statistical screening at the omics level, and hardly provided in-depth clues for potential functions of altered miRNAs.

Polybrominated diphenyl ethers (PBDEs), a class of brominated flame retardants, have been recognized as ubiquitous environmental pollutants primarily releasing from a wide variety of E-waste recycling sites [11,12]. Due to the high stability and lipophilicity, PBDEs could be highly accumulated in biota and therefore have posed serious ecological and health risks to the local environment and residents [13–15]. Our previous studies have associated motor hypoactivity of zebrafish larvae with some BDE47-responsive transcripts which were enriched in the processes of visual perception and bone development [16,17]. Then the regulatory factors behind gene expressions in toxic effects were the next focus we concerned.

Since deep sequencing provides a superior approach to microarray in high-throughput screening, miRNA-Seq was adopted to determine miRNA expression profiles of zebrafish larvae after a representative PBDE member, BDE47 exposure (low concentration exposure of 5 μg/L BDE47 and high concentration exposure of 500 μg/L BDE47, respectively). We designed multiple data analysis approaches to improve analysis results, for example, miRNA-mRNA negative correlation after target gene prediction for reducing false positives, miRNA-enrichment network for targeting miRNAs to their potential functions. Through the combination of these approaches, our study found BDE47 altered miRNA profiling of zebrafish larvae in response to transcriptomic changes, proposed several miRNAs including miR-735 and -301b relevant to BDE47-induced effects, and also identified two novel miRNAs which may exert their key functions. The strategy could be expected to apply in miRNA profiling analysis of wildlife facing various environmental risks.

## Results and Discussions

### Characterization of the miRNA profiling of zebrafish larvae

**Fig 1:**
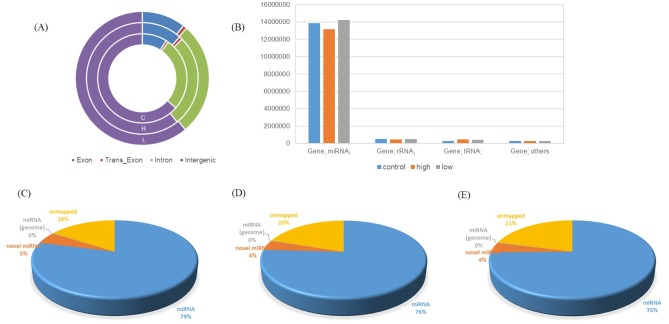
The quality information of sequencing. (A) The gene structure distributions. (B) The proportions of various type of RNAs. (C) Mapping status of vehicle control group. (D) Mapping status of 5 μg/L BDE47 group. (E) Mapping status of 500 μg/L BDE47 group.

The raw sequencing data are available at the Gene Expression Omnibus database (GSE84845). From Fig 1, although miRNA occupied only a small proportion of total RNA, the pre-treatment processes achieved preferable miRNA enrichment efficiencies to ensure the reliability of sequencing. The rates of mapping to zebrafish miRNA were all above 75%, and predicted miRNAs accounted for about 4–5%. Two BDE47-treated groups had a bit more unmapped clean reads than the control, which was a part of BDE47’s effects were unable to deal with at present. This phenomenon that still many novel miRNAs could not be mapped into validated zebrafish miRNAs or even any known genomes was primarily contributed to the deficiency of information in zebrafish miRNA database. For this reason, particular attentions should be required to balance the statistical accuracy and information sufficiency during our analysis.

### The significant effects of BDE47 on miRNA expressions

**Fig 2:**
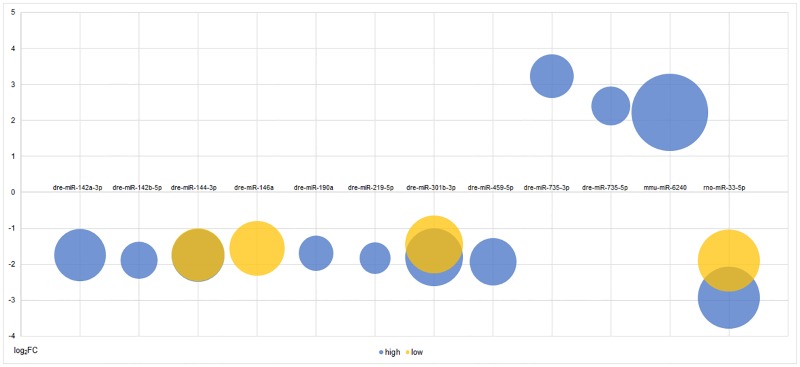
The fold changes and predicted target gene amounts of differentially expressed miRNAs under BDE47 treatments. The locations of bubble centers indicated fold changes of the miRNAs, and the sizes of bubbles indicated predicted target gene amounts. Blue: high concentration exposure; yellow: low concentration exposure.

Totally more than 3,000 of validated and predicted miRNAs had a *p*-value less than 0.05, in which 28 of them passed the additional criteria of FC > 2 and FDR < 0.05. Considering the reliability of predicted miRNAs for subsequent analysis, those successfully mapped to known mammalian genomes with remarkable expression were taken into account. According to this criteria, only two predicted miRNAs were regarded as differentially expressed, which were mapped to rodent rno-miR-33-5p and mmu-miR-6240 respectively. Totally, the final results included 9 downregulated miRNAs (dre-miR-142a-3p, dre-miR-142b-5p, dre-miR-144-3p, dre-miR-146a, dre-miR-190a, dre-miR-219-5p, dre-miR-301b-3p, dre-miR-459-5p and rno-miR-33-5p) and 3 upregulated miRNAs (dre-miR-735-3p, dre-miR-735-5p and mmu-miR-6240). Among them, dre-miR-146a only altered in low-concentration BDE47-treated larvae. Their expression changes and predicted target gene amounts were shown as Fig 2. The FCs of miRNAs were generally small under different BDE47 concentrations unlike the results of RNA-Seq [17], suggesting miRNAs possibly had a smoother expression change than mRNAs. Furthermore, miRNA tended to exert their functions while high concentration of BDE47 exposure, although the reasons still remained uncovered.

**Fig 3:**
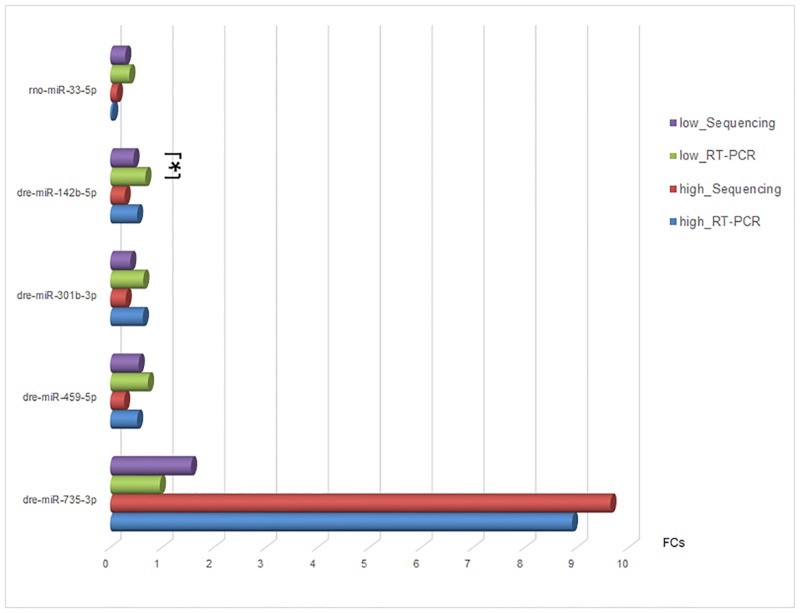
The quantitative miRNA expressions using sequencing and RT-PCR. The asterisk indicated only for dre-miR-142b-5p in low concentration BDE47 treatment, the result of statistical significance by RT-PCR determination was inconsistent with miRNA sequencing.

To validate the deep sequencing data, we used RT-PCR to assay the expression levels of five selected zebrafish miRNAs: miR-735-3p, -301b-3p, -142b-5p, -459-5p and putative zebrafish miR-33-5p. For predicted zebrafish miR-33, PCR could also confirm its actual existence in zebrafish. The results showed that expression variations of miRNAs by RT-PCR analysis were generally consistent with the sequencing (Fig 3).

### The miRNA-involving biological functions affected by BDE47

**Fig 4:**
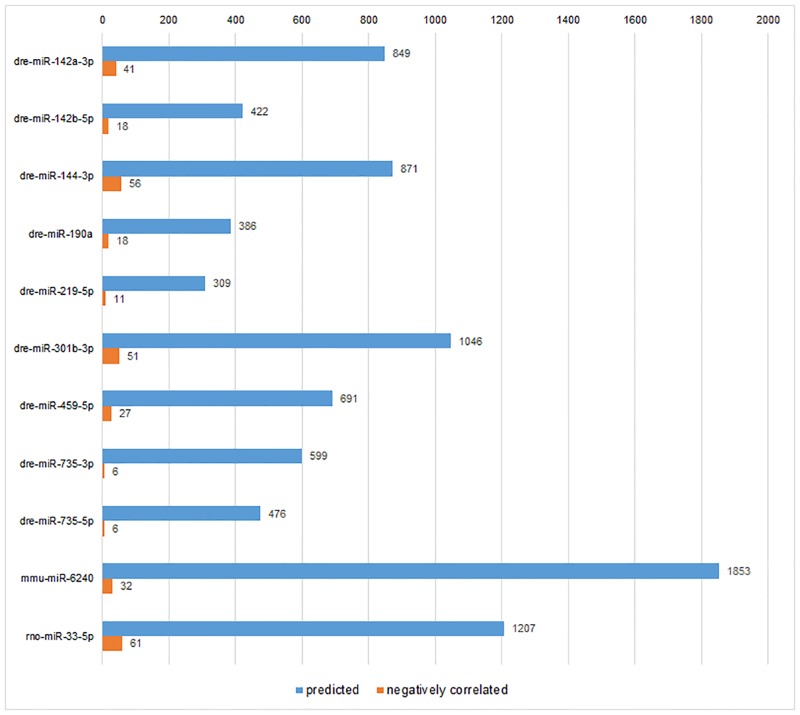
The comparison of numbers of predicted and negatively-correlated target genes in the group of high concentration of BDE47.

Because the biological roles of miRNAs must be achieved by their target genes, the required step before functional enrichment analysis of miRNA-Seq was inevitably target gene prediction. Our strategy here was combing miRanda prediction algorithm with miRNA-mRNA negative correlation. The miRanda prediction algorithm of target genes usually obtained more outputs than other common algorithms, like TargetScan and PicTar, while with higher FDR [18]. Compared with the estimation that one single human miRNA regulates ~200 human genes [19], the amounts of predicted target genes per miRNA in Fig 2 were averagely ~800, which suggested there were abundant false positives in prediction results. Consequently, miRNA-mRNA negative correlation was employed to reduce false positives obtained from miRanda. The negative correlation is actually intersecting predicted target genes of differentially expressed miRNAs and the differentially expressed mRNAs with opposite direction [20]. The transcriptomic data (GSE59968) were from our RNA-Seq of zebrafish larvae by the same treatment conditions. After negative correlation, the magnitudes of target genes were effectively controlled (Fig 4) and suitable for using in subsequent enrichment analysis.

**Fig 5:**
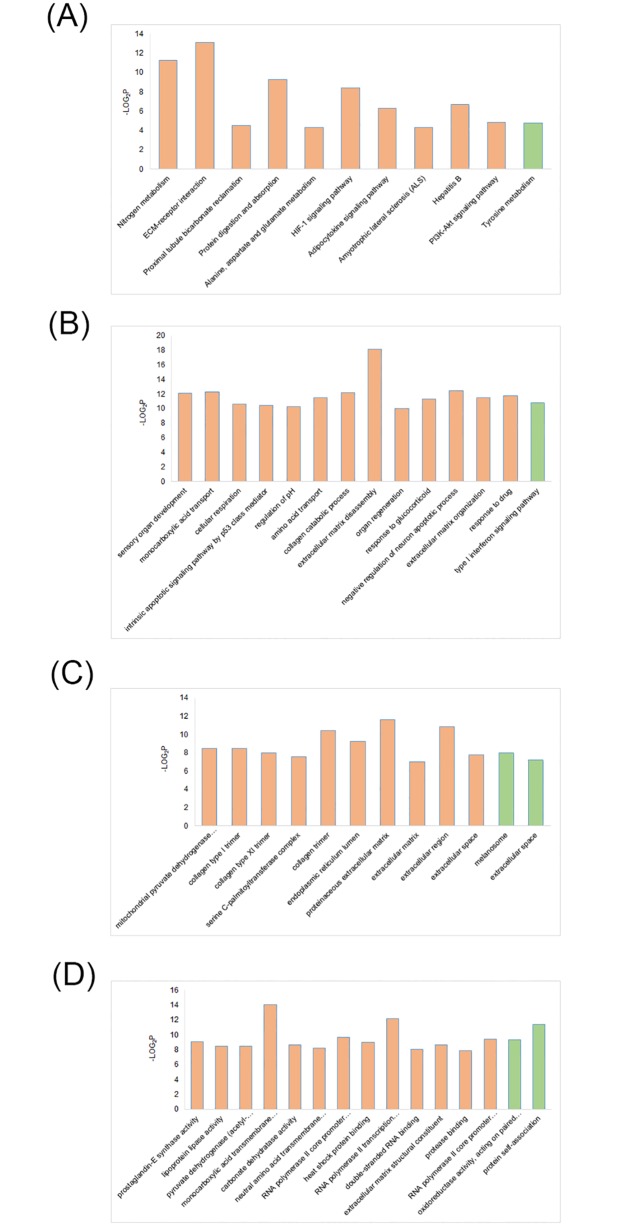
The most significantly altered enriched terms based on GO definitions and KEGG pathway definitions. (A) KEGG pathway terms. (B) GO BP terms. (C) GO CC terms. (D) GO MF terms. Yellow columns: significant in high concentration group; green columns: significant in high concentration group.

We performed functional enrichment analysis of the genes based on three domains: biological process (BP), molecular function (MF) and cellular component (CC) of the GO definition, as well as KEGG pathway definition. The list of the most significant terms (regardless of upregulation and downregulation) were shown in Fig 5. In high concentration of BDE47 group, the affected BP terms with high -log_2_*p* value and enrichment included sensory organ development, monocarboxylic acid transport, and collagen catabolic process. The results of enrichment analysis were similar with those in RNA-Seq [17], reflecting the involvement of miRNAs in these processes. The most outstanding characteristic in CC domain was several collagen trimer-related terms. Because of the relative simple structures, KEGG pathway analysis acquired fewer significantly differential terms. In addition, low concentration of BDE47 exposure had quite limited impacts on larvae.

### The construction of miRNA-enrichment term network

**Fig 6:**
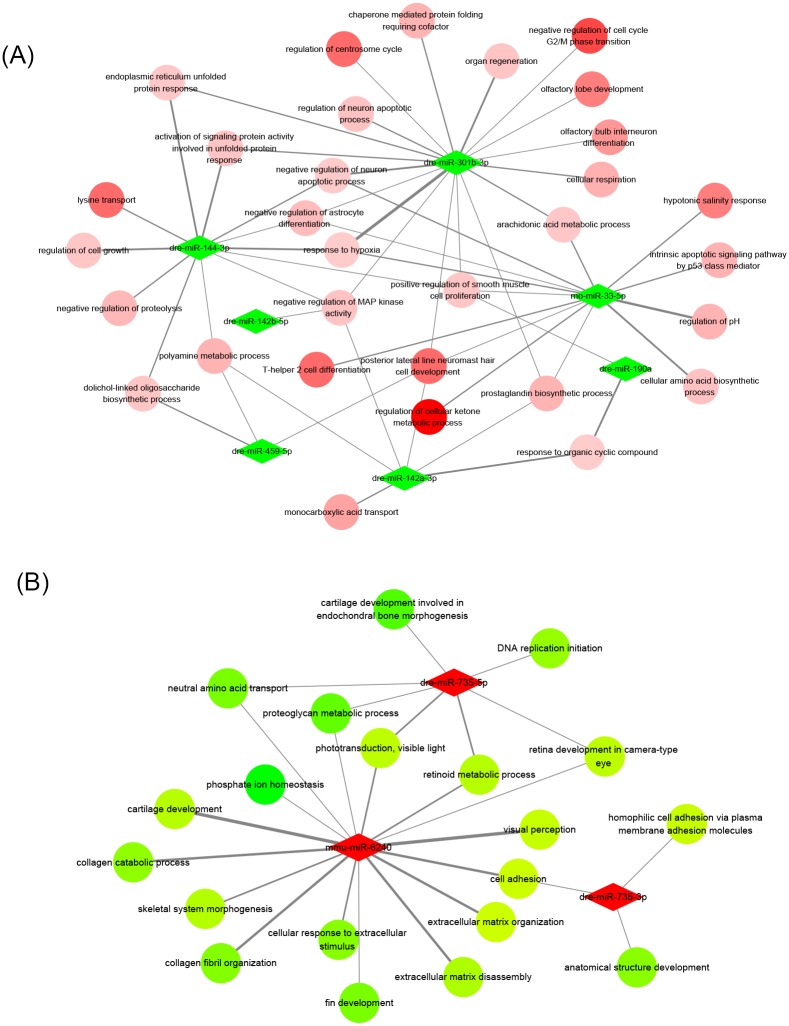
The miRNA-BP term network of BDE47-induced biological effects. (A) downregulated miRNAs. (B) upregulated miRNAs. Green: downregulated; red: upregulated. The color depths of circular nodes indicated the enrichment values of BP terms by functional enrichment analysis. The widths of edges indicated the gene numbers in BP terms regulated by source miRNAs.

miRNA target network which connected pivotal miRNAs and mRNAs based on their regulative relations (S1 Fig) was a common tool for data visualization in miRNA profiling analysis. Whereas such type of network was frequently challenged due to hardly explicitly reflect which specific biological functions the changed miRNAs could possibly exert influence on. Meanwhile, massive unenriched mRNAs often led to poor visualization of network. We therefore constructed miRNA-enrichment term interaction networks to overcome the above disadvantages. Fig 6 selected BP terms as samples, and networks based on other definitions could be seen in S2 and S3 Figs. In present networks, the relevance between miRNA and biological functions was represented by the “score” value (the size of edge between miRNA and enrichment term in Fig 6), and the value referred to the numbers of target genes from one single miRNA. The outputs of different networks could be slightly influenced by their different term definitions. In general, dre-miR-301b, -142a, -144, and rno-miR-33, were highly focused in network of downregulated miRNAs, while dre-miR-735 and mmu-miR-6240 were in network of upregulated miRNAs. Fig 6 also revealed the potential roles of upregulated miRNAs in BDE47-induced phenotypes.

### Potential critical roles of miRNAs in BDE47-related toxicity

Our constructed networks helped us to identify several candidate miRNAs in the biological effects induced by BDE47. In upregulated miRNAs, non-conservative Dicer-independent miR-735 was especially worth noting, which mostly accumulated in central nervous system although its distribution was known throughout the whole body of zebrafish [21]. Significant expression changes of miR-735 were also found in ethanol- and PFOS-exposed zebrafish larvae [22,23], reflecting this expression change was somehow universal. The functions of miR-735 could not be interpreted previously due to the lack of information. Here we addressed its potential characters in zebrafish neural and sensory development, which probably served as an indicator of pollutants exposure.

Unlike upregulated, downregulated miRNAs had more amounts and relatively even expression changes. One of them, miR-301b, belonged to miR-130 family, which was regarded as an oncogene and abnormally expressed in lung, breast and many other cancers [24–26]. MiR-301 also regulated the expression of key proteins involved in cholesterol homeostasis and blood lipid levels [27], consistent with what was indicated in S3 Fig. Although the research realm of miR-142a centered on cardiovascular and immune system [28,29], evidences suggested its renal-associated actions [30]. In addition, miR-142a was major constituent of mitochondrion-enriched miRNAs in response to nerve tissue injury [31]. The functions of downregulated miRNAs from our analysis were more uncertain than upregulated ones, and further investigation and validation were needed.

Finally, we identified two novel miRNAs, which were mapped into rodent miRNAs rno-miR-33-5p and mmu-miR-6240, and established their importance in BDE47’s toxicity by functional analysis. The existed information about mmu-miR-6240 was still inadequate, and it was estimated to exert functions in zebrafish neural and skeletal development based on our analysis, however its expression amounts were far less than miR-735. The utmost concern about miR-33 was its ability to regulate cellular cholesterol transport and maintain plasma HDL levels [32], which highly related with the physiological role of miR-301b. In our results, its potential functions in metabolism, neuron apoptosis, and stress response were identified.

A growing consensus is that the analysis of miRNA and various other regulatory factors in gene expression events should be integrated with their target genes [33]. The integration also aggravated the dependence on annotation integrity because even for those non-mammalian model organisms like zebrafish, they have quite incomplete miRNA database. On the other side, genes were unable to indicate actual biological effects unless they were functionally enriched. Considering miRNA-mRNA network and functional enrichment were commonly separated in current protocols, we hoped to construct a direct association between miRNAs and functional enrichment terms. By means of our analysis strategy, miR-735 was proposed to be main contributor participating in BDE47-induced effects of larval neural and sensory development.

The toxicity of PBDEs has been a research focus of ecotoxicology for many years. Following their disruption to animal thyroid hormones and neurobehavioral development, the representative BDE47 was found to impair visual perception and bone morphology. This study emphasized the regulatory roles of miRNAs corresponding to existed transcriptomic analysis of BDE47 [17]. The disruption of BDE47 on larval neural, sensory, and bone development was proved to be facilitated by several miRNAs, and the evidences of PBDEs’ effects on lipid metabolism of early zebrafish larvae began to emerge. Organisms seemed to have some common biological responses to pollutants, particularly in their early lives.

## Materials and Methods

### Chemicals and zebrafish exposure

BDE47 (purity > 99%) was purchased from Accustandard, Inc. (NewHaven, CT). Adult Wild-type Tuebingen zebrafish (*Danio rerio*) maintenance and embryo exposure were carried out according to our published protocols [16,17]. Briefly, 5–6 hpf normal embryos that reached the gastrula stage were selected by microscope and distributed randomly into 6-well plates. These embryos were exposed until 144 hpf. Each well contained about 50 embryos and 5 ml of BDE47 (0, 5, 500 μg/L, respectively) containing 0.1% DMSO (Amresco, Solon, OH). The concentration range was consistent with our previous study [17] for the convenience of quoting transcriptomic data. During the exposure period, the exposure solutions were renewed half daily. One replicate was used for deep sequencing and three replicates were used for RT-PCR.

### Ethics statement

The experimental protocols used in this study were approved by the Animal Ethics Committee of Tongji University.

### miRNA library construction and sequencing

Total RNA was extracted from 40–50 larvae by Trizol reagent (Invitrogen, Carlsbad, CA). The RNA quality was checked by Bioanalyzer 2200 (Aligent, Santa Clara, CA) and kept at -80°C. miRNAs were purified by miRNeasy Mini Kit (Qiagen, Germany) from RNAs with RIN > 8.0. The cDNA libraries for single-end sequencing were prepared using Truseq^™^ Small RNA sample prep Kit (Illumina, San Diego, CA) according to the manufacturer’s instructions. 1 μg small RNA was connected to add 3’ and 5’ adaptors and then enriched by PCR. Size selection was applied to obtain the 145–160 bp library sequence. TBS-380 Picogreen (Invitrogen, Carlsbad, CA) was introduced to quantify the library concentration. The sequencing was performed on Hiseq2000 platform (Illumina, San Diego, CA).

### miRNA mapping and novel miRNA prediction

The reads filtering towards the raw reads after sequencing were applied to achieve the clean data following the criteria: i) 30% base quality < 20, ii) read length < 17 bp, and iii) adaptor sequence. The clean data were mapped to zebrafish miRNA database (miRBase v21.0), and zebrafish genome (Zv10 assembly) to discover novel miRNAs using miRDeep algorithm [34]. The remaining reads were sequentially mapped to the rat, mouse, and human miRNA database (miRBase v21.0) for novel miRNA supplement. Burrows-Wheeler Aligner mapping software was utilized to achieve the novel miRNA expression.

### miRNA differential expression and target gene prediction

EB-Seq package [35] was employed to discover the differentially expressed miRNAs based on their counts with the *p*-value and false discovery rate (FDR) analysis under the following criteria: i) Fold Change > 2 or < 0.5, and ii) *p*-value < 0.05, FDR < 0.05. We applied the Miranda target analysis to predict the miRNA target on zebrafish mRNA. To discover the potential miRNA target in this research, we filtered out the miRNA target without the negative correlation analysis between the mRNA and miRNA. The mRNA-Seq data used in negative correlation was from our previous study [17], and the detailed remapping methods could be seen in S1 Text.

### Functional enrichment analysis

Gene ontology (GO) analysis was performed to facilitate elucidating the biological implications of unique genes in the significant or representative profiles of the differentially expressed gene in the experiment. We downloaded the GO annotations from NCBI (http://www.ncbi.nlm.nih.gov/), UniProt (http://www.uniprot.org/) and the Gene Ontology (http://www.geneontology.org/). Fisher’s exact test was applied to identify the significant GO categories and FDR was used to correct the *p*-values. Pathway analysis was adopted to find out the significant pathway of the differential genes according to KEGG database. We also used Fisher’s exact test to select the significant pathway, and the threshold of significance was defined by *p*-value and FDR, separately. All kinds of miRNA target networks were illustrated using Cytoscape 3.4.0.

### Real-time PCR validation

The quantitative expressions of larval miRNA were validated using Real-time PCR (RT-PCR) with All-in-One^™^ miRNA qRT-PCR Detection Kit (GeneCopoeia, Rockville, MD) according to the manufacturer's protocol. In brief, 2 μg of total RNA was reverse-transcribed to cDNA before RT-PCR. PCR were performed on an ABI Prism 7500 (Applied Biosystems, Foster City, CA) with the following program: 95°C for 10 min, and 35 cycles of 10 s at 95°C, 20 s at 60°C, and 30 s at 72°C. Human U6 snRNA was used as endogenous reference [9] and relative expression changes were calculated using the 2^-ΔΔCt^ method.

## Supporting Information

S1 Fig miRNA target network in BDE47-induced biological effects. miRNAs with their target genes based on sequence complementary. (A) downregulated miRNAs with upregulated target genes. (B) upregulated miRNAs with downregulated target genes.
(TIF)

S2 Fig The miRNA-CC term network of BDE47-induced biological effects. (A) downregulated miRNAs. (B) upregulated miRNAs. Green: downregulated; red: upregulated. The color depths of circular nodes indicated the enrichment values of CC terms by functional enrichment analysis. The widths of edges indicated the gene numbers in CC terms regulated by source miRNAs.
(TIF)

S3 Fig The miRNA-pathway term network of BDE47-induced biological effects. (A) downregulated miRNAs. (B) upregulated miRNAs. Green: downregulated; red: upregulated. The color depths of circular nodes indicated the enrichment values of pathway terms by functional enrichment analysis. The widths of edges indicated the gene numbers in pathway terms regulated by source miRNAs.
(TIF)

S1 Text Supplementary Methods: The remapping of RNA-Seq data (GSE59968). (PDF)
