# Supplementary figures and images for: The Regulatory Roles of MicroRNA in Effects of 2,2'4,4'-Tetrabromodiphenyl Ether (BDE47) on the Transcriptome of Zebrafish Larvae
Source: PLoS One. 2017 Jan 10;12(1):e0169599. doi: 10.1371/journal.pone.0169599 (PMC5225001; doi:10.1371/journal.pone.0169599)

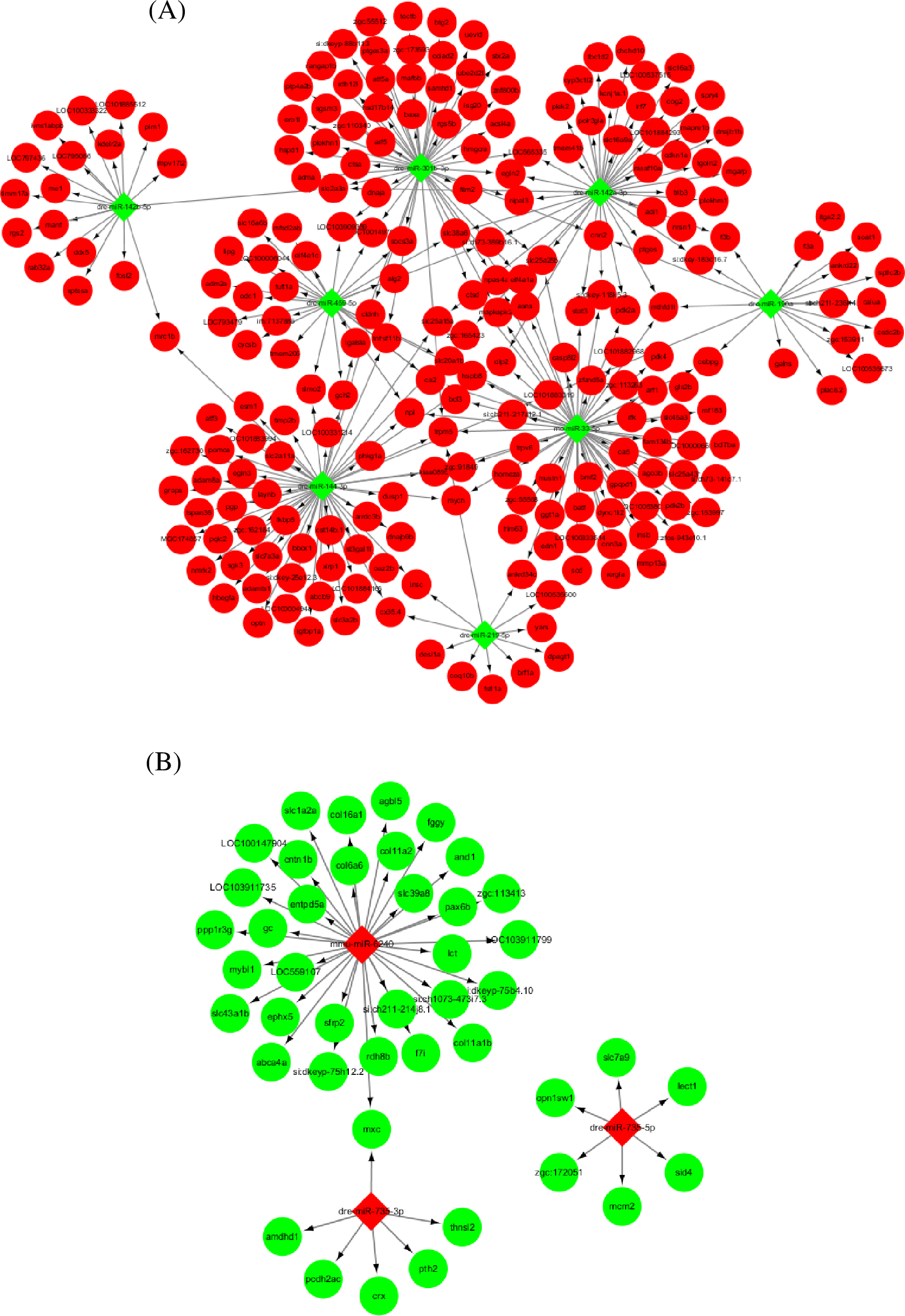

Supplement: S1 Fig — miRNAs with their target genes based on sequence complementary. (A) downregulated miRNAs with upregulated target genes. (B) upregulated miRNAs with downregulated target genes. (TIF) [file pone.0169599.s001.tif]

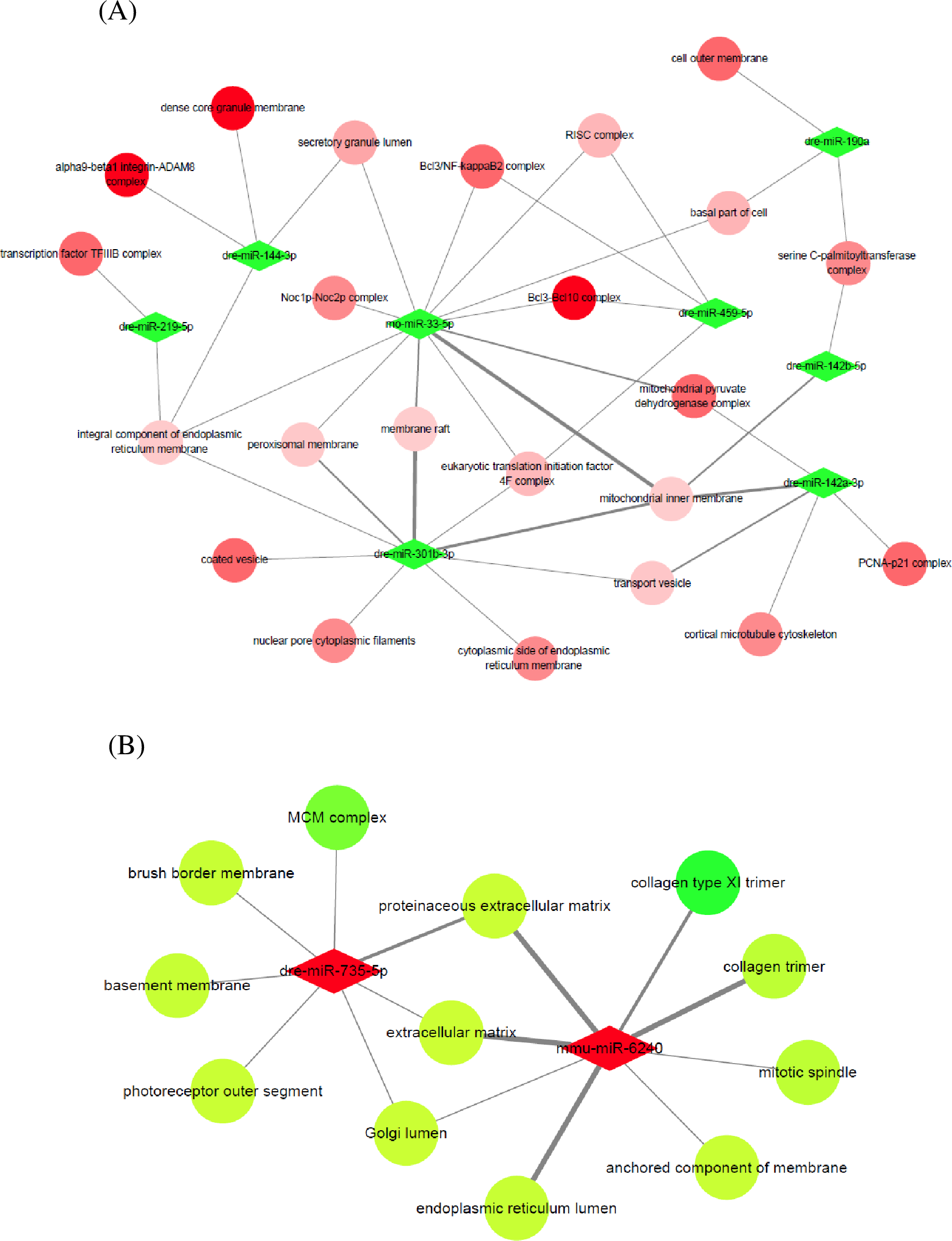

Supplement: S2 Fig — (A) downregulated miRNAs. (B) upregulated miRNAs. Green: downregulated; red: upregulated. The color depths of circular nodes indicated the enrichment values of CC terms by functional enrichment analysis. The widths of edges indicated the gene numbers in CC terms regulated by source miRNAs. (TIF) [file pone.0169599.s002.tif]

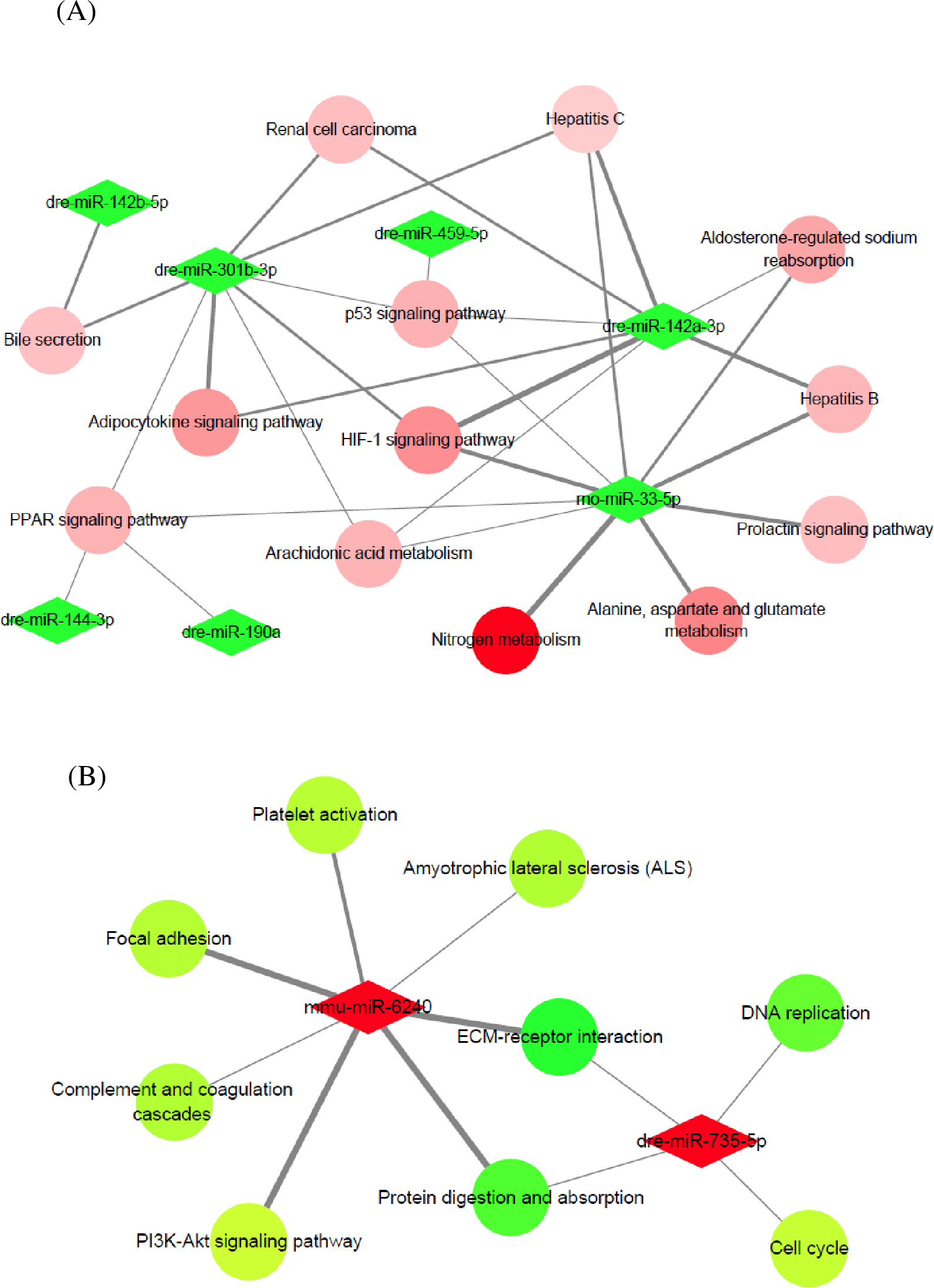

Supplement: S3 Fig — (A) downregulated miRNAs. (B) upregulated miRNAs. Green: downregulated; red: upregulated. The color depths of circular nodes indicated the enrichment values of pathway terms by functional enrichment analysis. The widths of edges indicated the gene numbers in pathway terms regulated by source miRNAs. (TIF) [file pone.0169599.s003.tif]
