# Supplementary material for: The Regulatory Roles of MicroRNA in Effects of 2,2'4,4'-Tetrabromodiphenyl Ether (BDE47) on the Transcriptome of Zebrafish Larvae
Source: PLoS One. 2017 Jan 10;12(1):e0169599. doi: 10.1371/journal.pone.0169599 (PMC5225001; doi:10.1371/journal.pone.0169599)
Supplement: S1 Text — (PDF) [file pone.0169599.s004.pdf]

Text S1 The remapping of RNA-Seq data (GSE59968).

The transcriptomic data (GSE59968) required by miRNA-mRNA negative correlation was from our previous study [1]. Because of the evolution of genome database, the data need be remapped to acquire recent annotation information. Before read mapping, clean reads were obtained from the raw reads by removing the adaptor sequences, reads with >5% ambiguous bases (noted as N) and low-quality reads containing more than 20 percent of bases with qualities of < 20. The clean reads were then aligned to zebrafish genome (version Zv10) using the Tophat program [2]. EB-Seq package [3] was applied to discover the differentially expressed mRNA based on mRNA counts with the *p*-value and FDR analysis [4] under the following criteria: i) fold change > 2 or < 0.5; ii) *p*-value < 0.05, FDR < 0.05. The results could be used in the analysis of negative correlation.

References

[1] T. Xu, J. Zhao, D.-Q. Yin, Q.-S. Zhao, B. Z. Dong, High-throughput RNA sequencing reveals the effects of 2,2',4,4'-tetrabromodiphenyl ether on retina and bone development of zebrafish larvae, BMC Genomics 16 (2015) 23.

[2] C. Trapnell, A. Roberts, L. Goff, G. Pertea, D. Kim, D.R. Kelley, H. Pimentel, S.L. Salzberg, J.L. Rinn, L. Pachter, Differential gene and transcript expression analysis of RNA-seq experiments with TopHat and Cufflinks, Nat. Protoc. 7 (2012) 562-578.

[3] N. Leng, J.A. Dawson, J.A. Thomson, V. Ruotti, A.I. Rissman, B.M.G. Smits, J.D. Haag, M.N. Gould, R.M. Stewart, C. Kendziorski, EBSeq: An empirical Bayes hierarchical model for inference in RNA-seq experiments, Bioinformatics 29 (2013) 1035-1043.

[4] Y. Benjamini, D. Drai, G. Elmer, N. Kafkafi, I. Golani, Controlling the false discovery rate in behavior genetics research, Behav. Brain Res. 125 (2001) 279-284.
